# Repetitive Transcranial Magnetic Stimulation Combined with Ginkgo Diterpene Lactone Meglumine Injection Recover Cognitive and Neurological Functions of Patients with Acute Ischemic Stroke

**DOI:** 10.62641/aep.v53i1.1676

**Published:** 2025-01-05

**Authors:** Mengwei Hao, Xuxia Wang, Tao Wei, Chao Sheng

**Affiliations:** ^1^Department of Neurology, Xuzhou Central Hospital, 221000 Xuzhou, Jiangsu, China; ^2^Department of Rehabilitation Medicine, Xuzhou Central Hospital, 221000 Xuzhou, Jiangsu, China

**Keywords:** repetitive transcranial magnetic stimulation, ginkgo diterpene lactone meglumine injection, acute ischemic stroke, lipoprotein-associated phospholipase A2, ischemia-modified albumin

## Abstract

**Background:**

Acute ischemic stroke (AIS) is a prevalent and challenging neurological condition associated with high mortality and morbidity rates. This study aimed to evaluate the therapeutic efficacy of repetitive transcranial magnetic stimulation (rTMS) combined with ginkgo diterpene lactone meglumine injection (GDLMI) on cognitive and neurological function recovery in patients with AIS.

**Methods:**

A total of 120 patients with AIS, admitted between January 2021 and January 2022, received rTMS combined with GDLMI after admission. Their cognitive and neurological functions were assessed using the Chinese version of the Montreal Cognitive Assessment (MoCA) and the National Institute of Health Stroke Scale (NIHSS) respectively before and after treatment. Additionally, serum levels of lipoprotein-associated phospholipase A_2_ (Lp-PLA_2_) and ischemia-modified albumin (IMA) were quantified. Statistical analyses were performed to elucidate potential correlations between Lp-PLA_2_ and IMA levels and clinical outcomes.

**Results:**

After treatment, patients with AIS exhibited significantly improved cognitive and neurological functions, increased MoCA score and decreased NIHSS score compared to those before treatment (*p* < 0.05). A linear correlation was observed between Lp-PLA_2_ and IMA levels and the recovery of cognitive function in AIS patients (*r* = –0.892/–0.764, *p* < 0.05). Before and after factor adjustment, Lp-PLA_2_ and IMA were identified as independent influencing factors for the efficiency in cognitive function recovery (*p* < 0.05). Similarly, Lp-PLA_2_ and IMA levels were linearly correlated with the recovery of neurological function in AIS patients (*r* = –0.887/–0.796, *p* < 0.05). Lp-PLA_2_ combined with IMA performed better than Lp-PLA_2_ or IMA alone in predicting the efficiency of rTMS plus GDLMI in promoting the cognitive and neurological function recovery (*p* < 0.05).

**Conclusions:**

rTMS combined with GDLMI can contribute to the cognitive and neurological function recovery in patients with AIS. Serum levels of Lp-PLA_2_ and IMA could serve as independent influencing factors for the efficiency in promoting cognitive and neurological function recovery.

## Introduction

Acute ischemic stroke (AIS) is a clinically common intractable disease with high mortality and morbidity rates. It has been found that AIS is often accompanied by varying degrees of cognitive and neurological impairment [1]. Consequently, early intervention is crucial for optimizing the recovery of cognitive and neurological functions. Ginkgo diterpene lactone meglumine injection (GDLMI) is a commonly used therapeutic agent in the treatment of AIS. It has been shown to facilitate the recovery of patients’ cognitive and neurological functions by attenuating the progression of brain injury [2]. However, clinical observations indicate that GDLMI alone demonstrates limited efficacy in clinical use. Repetitive transcranial magnetic stimulation (rTMS) is a non-invasive, well-tolerated, and efficacious cortical stimulation therapy that has demonstrated therapeutic potential in the treatment of AIS [3]. Nevertheless, the synergistic effects of rTMS combined with GDLMI on the improvement of cognitive and neurological functions of AIS patients remain largely unexplored. Moreover, the prognostic trajectory of these patients is significantly affected by the initial severity of the disease.

Lipoprotein-associated phospholipase A_2_ (Lp-PLA_2_) has been demonstrated to promote atherosclerosis, and its association with cognitive and neurological impairment in AIS has been verified [4]. rTMS has been shown to markedly modulate Lp-PLA_2_ activity [5]. Furthermore, Lp-PLA_2_ may serve as a biomarker for sub-acute stroke patients receiving treatment with GDLMI [6]. Elevated levels of ischemia-modified albumin (IMA), a kind of serum albumin, are frequently indicative of AIS progression [7]. However, the influence of rTMS or GDLMI on IMA expression remains to be elucidated.

This study aimed to elucidate the potential relationship between the combined application of rTMS and GDLMI and the levels of Lp-PLA_2_ and IMA in the context of cognitive and neurological function recovery in patients with AIS. The investigation sought to provide valuable clinical evidence for future treatment.

## Materials and Methods

### Subjects

The study received ethical approval (No. JSXZH2021403) and was conducted in accordance with the principles of the Declaration of Helsinki. Informed consent was obtained from all participants before enrollment. The study cohort comprised 120 patients with AIS who were treated at Xuzhou Central Hospital between January 2021 and January 2022. They included 68 males and 52 females, with a mean age of 56.84 ± 5.19 years. Inclusion criteria were as follows: meeting the clinical diagnostic criteria for AIS [8]; experiencing first-onset AIS; voluntary participation and provision of written informed consent. Exclusion criteria encompassed: comorbid cerebral hemorrhage, massive cerebral infarction, transient ischemic attack, mental illness or benign/malignant tumors, and impaired communication abilities.

### rTMS Combined with GDLMI

Patients received rTMS combined with GDLMI immediately after hospitalization. rTMS was administered using NS5000 transcranial magnetic stimulator (Wuhan Yiruide Medical Equipment New Technology Co., Ltd., Wuhan, China). The stimulation coil was placed over the dorsolateral region of the left frontal lobe, with the nasal-occipital line on the locating cap in the median head, and the CZ point at the midpoint of the connection between the posterior occipital tuberosity and the eyebrow center. The coil was tangent to the skull surface, with the stimulation focus centered at the intersection of the two circles. Stimulation parameters were set at 10 Hz, with 3 s/time with an interval of 30 s, for a total duration of 20 min. GDLMI treatment consisted of a daily intravenous injection of 5 mL GDLMI (Cat. No. Z20120024; Jiangsu Kanion Pharmaceutical Co., Ltd., Lianyungang, China) diluted with 250 mL of 0.9% sodium chloride. The combined treatment was administered for 14 consecutive days.

### Assessment of Cognitive Function

Cognitive function assessment was conducted before and after treatment by two independent, qualified, and experienced evaluators using the validated Chinese version of the Montreal Cognitive Assessment (MoCA). The MoCA comprised 11 items across 8 domains, including executive function, attention and concentration, and language, among others [9]. The score ranges from 0 to 30 points, with a higher score indicating a superior cognitive function. A score <26 points is indicative of cognitive impairment. Following the administration of rTMS combined with GDLMI, participants were divided into two cohorts: a cognitive impairment group and a normal cognitive function group according to the presence or absence of cognitive impairment.

### Assessment of Neurological Function

Neurological function was evaluated before and after treatment by two independent and experienced clinicians using the National Institute of Health Stroke Scale (NIHSS). This validated assessment tool comprised 11 items, including measures of consciousness and limb movement [10]. The NIHSS score ranges from 0 to 42 points, with higher scores indicating more severe neurological impairment. A score ≥1 is indicative of neurological impairment. Following rTMS combined with GDLMI, patients were divided into two cohorts: a neurological impairment group and a normal neurological function group according to the presence or absence of neurological impairment.

### Detection of Serum Levels of Lp-PLA_2_ and IMA

Fasting venous blood samples (3 mL) were collected from each patient in the morning and centrifuged to obtain the serum. Serum levels of Lp-PLA_2_ and IMA were quantified using enzyme-linked immunosorbent assay (ELISA) kits (Invitrogen, Waltham, MA, USA, catalog number: EH304RB; MyBioSource, USA, catalog number: MBS263569). Absorbance measurements were performed using a multifunctional microplate reader (Mithras LB940, Berthold Technologies, Bad Wildbad, Germany).

### Observation Indices

(1) Cognitive and neurological functions were assessed before and after treatment. (2) Demographic and clinical data were collected for four distinct groups: cognitive impairment group, normal cognitive function group, neurological impairment group, and normal neurological function group. Variables included age, sex, body mass index (BMI), medical history (hypertension, coronary heart disease, *etc*.), familial stroke history, smoking and alcohol consumption patterns, laboratory parameters [fasting blood glucose (FBG) and total cholesterol (TC)], duration from onset to admission and NIHSS score at admission. Comparative analyses were conducted to evaluate differences in Lp-PLA_2_ and IMA levels between the respective subgroups. (3) Logistic regression analysis was employed to evaluate the correlations between Lp-PLA_2_ and IMA levels and the efficiency of combined rTMS and GDLMI in promoting cognitive and neurological function recovery among patients with AIS. (4) The predictive efficacy of Lp-PLA_2_ and IMA for the efficiency of rTMS plus GDLMI in promoting cognitive and neurological function recovery in AIS patients was analyzed using receiver operating characteristic (ROC) curves.

### Statistical Analysis

Statistical analysis was conducted using SPSS version 26.0 software (IBM Inc., Armonk, NY, USA). The Kolmogorov-Smirnov test was employed to assess the normality of distribution, while Levene’s test was used to evaluate the homogeneity of variance. Continuous variables with normal distribution were expressed as mean ± standard deviation ($\bar{x}$ ±*s*) and analyzed using one-way F analysis or independent samples *t*-test, as appropriate. Non-normally distributed data underwent natural logarithmic transformation and were presented as a median and interquartile range [*M(Qn)*], with subsequent analysis using non-parametric tests. Categorical variables were described as [n (%)] and compared between two groups using the χ^2^ test. The correlations between Lp-PLA_2_ and IMA levels and the efficiency of combined rTMS and GDLMI in promoting cognitive and neurological function recovery in AIS patients were subjected to logistic regression analysis. Pearson’s correlation analysis was conducted. The predictive efficacy of Lp-PLA_2_ and IMA for the efficiency of the rTMS plus GDLMI in promoting cognitive and neurological function recovery in AIS patients was analyzed using ROC curves. Statistical significance was set at *p* < 0.05. All statistical tests were conducted as two-tailed analyses, with a significance level of α = 0.05.

## Results

### Cognitive and Neurological Functions of AIS Patients before and after rTMS Combined with GDLMI

After treatment, patients with AIS exhibited significantly improved cognitive and neurological functions, increased MoCA score and decreased NIHSS score compared to those before treatment (*p* < 0.05) (Table 1). Among the 120 patients, 18 and 25 patients still had mild cognitive impairment and mild neurological impairment, respectively.

**Table 1.** **Cognitive and neurological functions of 120 AIS patients before and after rTMS combined with GDLMI [($\bar{x}$ ±*s*), point]**.

	Before treatment	After treatment	*t*	*p*
MoCA score	18.32 ± 1.29	27.93 ± 4.31	23.400	<0.001
NIHSS score	9.32 ± 1.19	2.64 ± 0.28	59.860	<0.001

AIS, acute ischemic stroke; rTMS, repetitive transcranial magnetic stimulation; GDLMI, ginkgo diterpene lactone meglumine injection; MoCA, Montreal Cognitive Assessment; NIHSS, National Institute of Health Stroke Scale.

### General Data, Lp-PLA_2_ and IMA of Cognitive Impairment and Normal Cognitive Function Groups

The proportions of patients with history of hypertension and coronary heart disease, family history of stroke, and smoking and drinking history, the levels of FBG, TC, Lp-PLA_2_ and IMA, and the NIHSS score at admission were lower, and the duration from onset to admission was shorter in the normal cognitive function group than those in the cognitive impairment group (*p* < 0.05) (Table 2).

**Table 2.** **General data, Lp-PLA_2_ and IMA of cognitive impairment and normal cognitive function groups**.

	Cognitive impairment group (n = 18)	Normal cognitive function group (n = 102)	*t/χ^2^*	*p*
Gender (male/female)	9/9	59/43	0.383	0.536
Age (year)	56.78 ± 6.43	56.99 ± 6.52	0.126	0.900
BMI (kg/m^2^)	23.41 ± 2.97	23.50 ± 2.86	0.122	0.903
Hypertension (Yes/No)	11/7	30/72	6.835	0.009
Coronary heart disease (Yes/No)	13/5	28/74	13.635	<0.001
Family history of stroke (Yes/No)	15/3	12/90	44.942	<0.001
Smoking and drinking history (Yes/No)	16/2	31/67	20.685	<0.001
FBG (mmol/L)	8.65 ± 0.43	6.12 ± 0.74	14.060	<0.001
TC (mmol/L)	5.64 ± 0.74	4.00 ± 0.32	15.720	<0.001
Duration from onset to admission (h)	6.34 ± 0.53	4.24 ± 0.65	12.950	<0.001
NIHSS at admission (point)	11.97 ± 1.08	8.01 ± 0.78	18.660	<0.001
Lp-PLA_2_ (µg/L)	261.96 ± 25.89	68.77 ± 7.92	61.650	<0.001
IMA (U/mL)	123.54 ± 13.67	78.65 ± 9.31	17.460	<0.001

Lp-PLA_2_, lipoprotein-associated phospholipase A_2_; IMA, ischemia-modified albumin; BMI, body mass index; FBG, fasting blood glucose; TC, total cholesterol.

### Correlations of Lp-PLA_2_ and IMA with Cognitive Function Recovery in AIS Patients

**Fig. 1.**
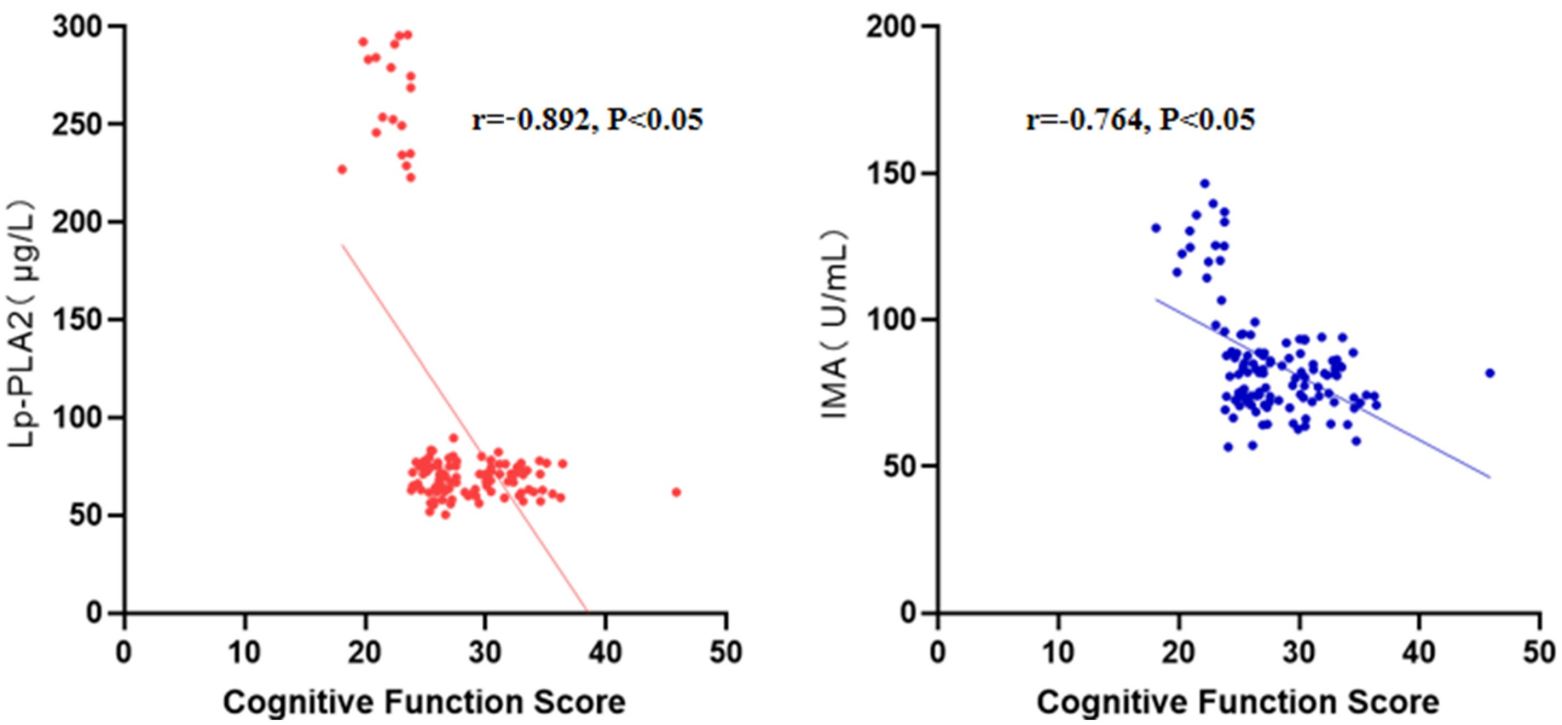
**Correlations of Lp-PLA_2_ and IMA with cognitive function recovery in AIS patients**.

The correlation analysis revealed a statistically linear association between Lp-PLA_2_ and IMA levels and the recovery state of cognitive function in patients with AIS (*r* = –0.892/–0.764, *p* < 0.05) (Fig. 1).

### Associations of Lp-PLA_2_ and IMA with the Efficiency of rTMS Combined with GDLMI in Promoting Cognitive Function Recovery in AIS Patients

Before and after factor adjustment, Lp-PLA_2_ and IMA were proven to be independent influencing factors for the efficiency of rTMS combined with GDLMI in promoting cognitive function recovery (*p* < 0.05) (Table 3).

**Table 3.** **Associations of Lp-PLA_2_ and IMA with the efficiency of rTMS combined with GDLMI in promoting cognitive function recovery in AIS patients before and after factor adjustment**.

	Before adjustment					After adjustment				
	β	SE	Wald*/$\chi^{2}$*	OR (95% CI)	*p*	β	SE	Wald*/$\chi^{2}$*	OR (95% CI)	*p*
Lp-PLA_2_	1.083	0.323	11.242	2.954 (1.568–5.563)	0.001	1.024	0.439	5.441	2.784 (1.178–6.583)	0.020
IMA	1.437	0.401	12.842	4.208 (1.918–9.235)	<0.001	1.304	0.535	5.941	3.684 (1.291–10.513)	0.015

The analysis was adjusted for several factors, including medical history of hypertension and coronary heart disease, family history of stroke, smoking and alcohol consumption habits, FBG, TC, duration from onset to admission, and NIHSS score at admission.

### General Data, Lp-PLA_2_ and IMA of Neurological Impairment and Normal Neurological Function Groups

The proportions of patients with history of hypertension and coronary heart disease, family history of stroke, and smoking and drinking history, the levels of FBG, Lp-PLA_2_ and IMA, and the NIHSS score at admission were lower, and the duration from onset to admission was shorter in the normal neurological function group than those in the neurological impairment group (*p* < 0.05) (Table 4).

**Table 4.** **General data, Lp-PLA_2_ and IMA of neurological impairment and normal neurological function groups**.

	Neurological impairment group (n = 25)	Normal neurological function group (n = 95)	*t/χ^2^*	*p*
Gender (male/female)	12/13	56/39	0.967	0.326
Age (year)	56.56 ± 5.23	56.71 ± 5.34	0.126	0.900
BMI (kg/m^2^)	23.14 ± 2.51	23.16 ± 2.56	0.034	0.972
Hypertension (Yes/No)	16/9	25/70	12.495	<0.001
Coronary heart disease (Yes/No)	18/7	23/72	28.044	<0.001
Family history of stroke (Yes/No)	10/15	17/78	5.546	0.019
Smoking and drinking history (Yes/No)	19/6	28/67	17.981	<0.001
FBG (mmol/L)	8.92 ± 1.02	6.43 ± 0.52	16.950	<0.001
TC (mmol/L)	4.72 ± 0.36	4.77 ± 0.45	0.514	0.609
Duration from onset to admission (h)	6.12 ± 0.93	4.00 ± 0.43	16.590	<0.001
NIHSS at admission (point)	12.55 ± 1.34	8.43 ± 0.58	23.030	<0.001
Lp-PLA_2_ (µg/L)	253.91 ± 29.03	65.93 ± 5.12	60.310	<0.001
IMA (U/mL)	119.28 ± 15.43	76.91 ± 6.41	20.920	<0.001

### Correlations of Lp-PLA_2_ and IMA with Neurological Function Recovery in AIS Patients

**Fig. 2.**
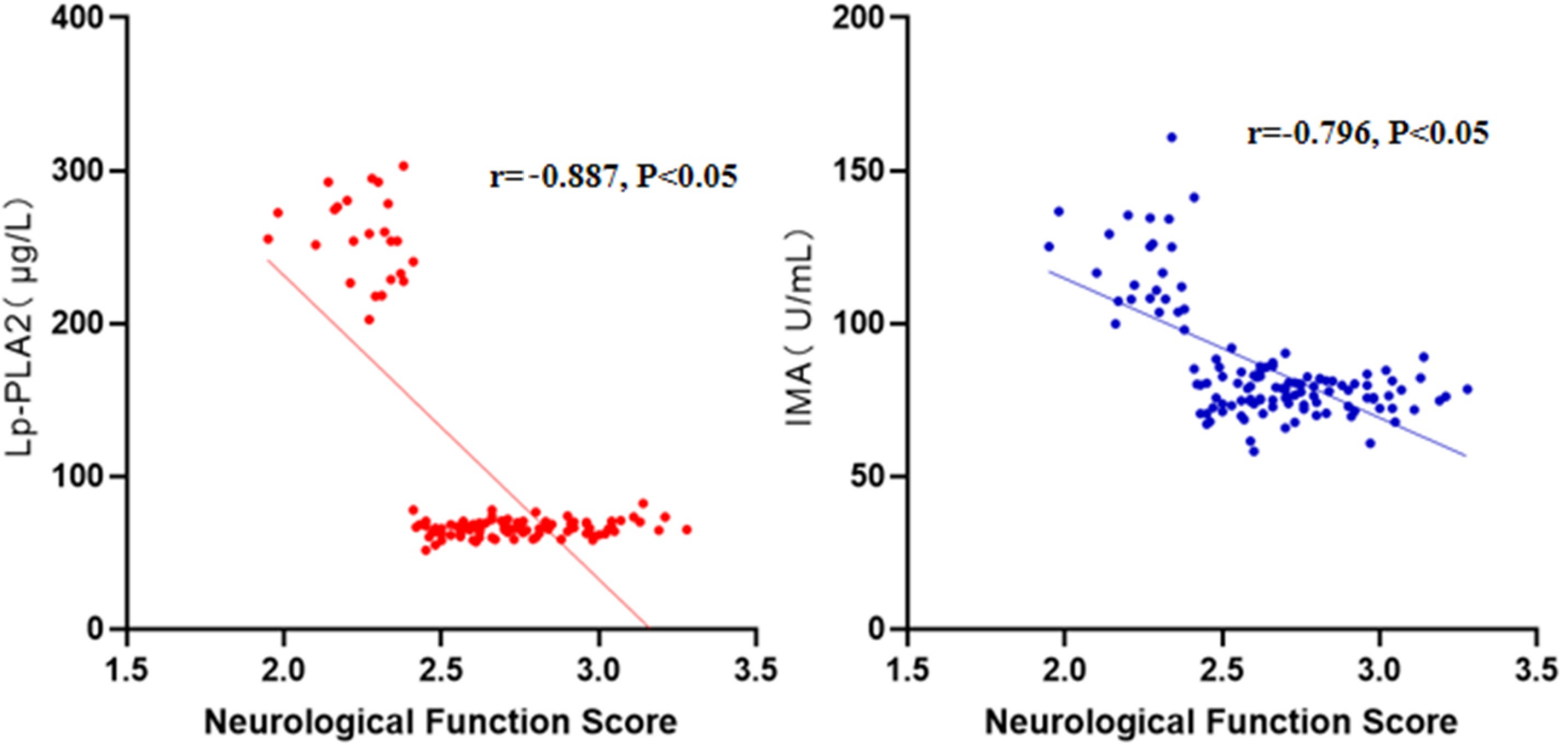
**Correlations of Lp-PLA_2_ and IMA with neurological function recovery in AIS patients**.

The results of correlation analysis showed that Lp-PLA_2_ and IMA levels were linearly correlated with the recovery state of neurological function in AIS patients (*r* = –0.887/–0.796, *p* < 0.05) (Fig. 2).

### Associations of Lp-PLA_2_ and IMA with the Efficiency of rTMS Combined with GDLMI in Promoting Neurological Function Recovery in AIS Patients

Before and after factor adjustment, Lp-PLA_2_ and IMA were demonstrated to be independent influencing factors for the efficiency of rTMS combined with GDLMI in promoting neurological function recovery (*p* < 0.05) (Table 5).

**Table 5.** **Associations of Lp-PLA_2_ and IMA with the efficiency of rTMS combined with GDLMI in promoting neurological function recovery in AIS patients before and after factor adjustment**.

	Before adjustment					After adjustment				
	β	SE	Wald*/χ^2^*	OR (95% CI)	*p*	β	SE	Wald*/χ^2^*	OR (95% CI)	*p*
Lp-PLA_2_	1.145	0.451	6.446	3.142 (1.298–7.606)	0.011	0.989	0.326	9.204	2.689 (1.419–5.093)	0.002
IMA	1.443	0.563	6.569	4.233 (1.404–12.762)	0.010	1.269	0.496	6.546	3.557 (1.346–9.404)	0.011

The analysis was adjusted for different factors, including medical history of hypertension and coronary heart disease, family history of stroke, smoking and alcohol consumption history, FBG, duration from symptom onset to hospital admission, and NIHSS score on admission.

### Predictive Efficacy of Lp-PLA_2_ and IMA for the Efficiency of rTMS Plus GDLMI in Promoting the Cognitive and Neurological Function Recovery in AIS Patients Using ROC Curves

**Fig. 3.**
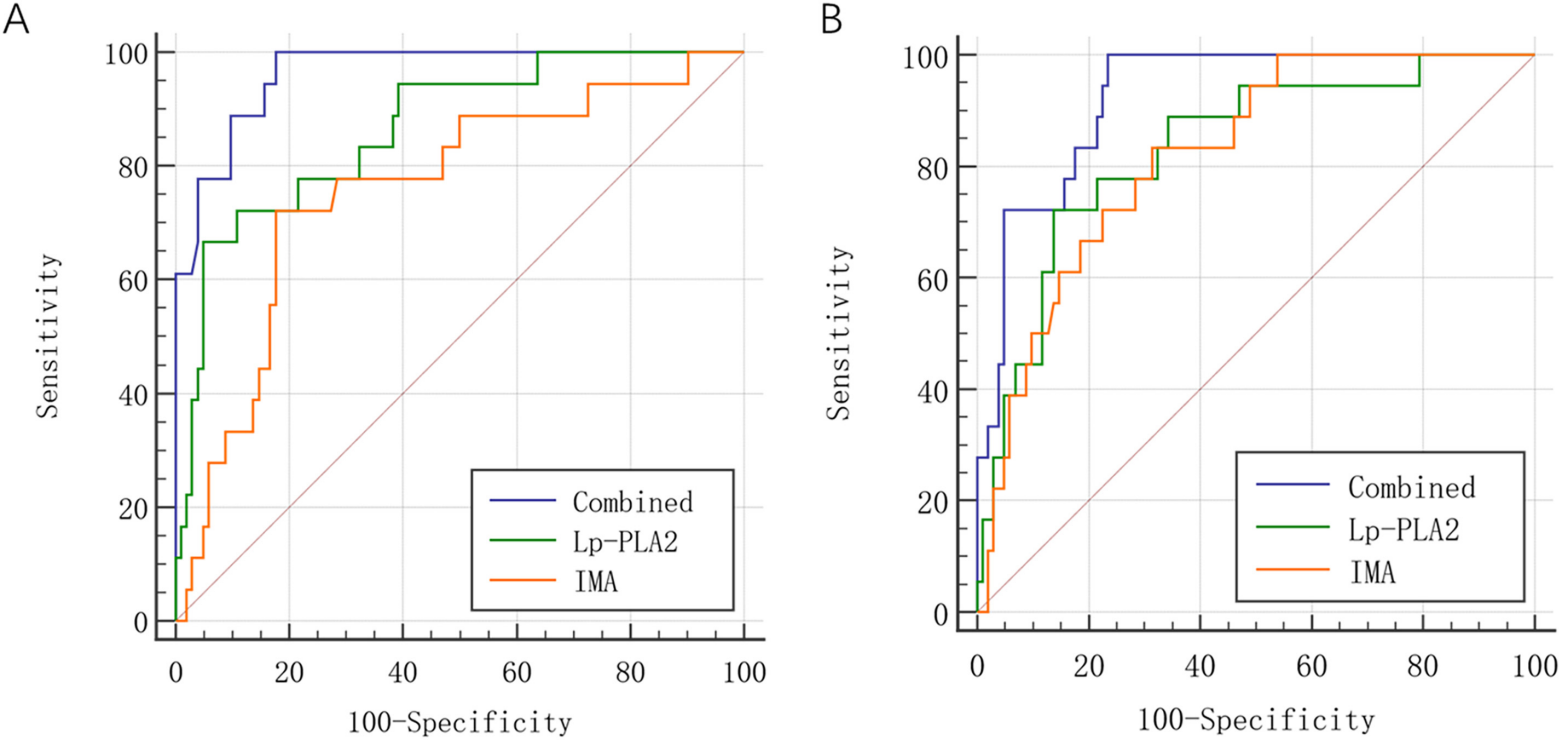
**Receiver operating characteristic (ROC) curves for predictive efficacy of Lp-PLA_2_ and IMA for efficiency of rTMS plus GDLMI in promoting (A) the cognitive and (B) neurological function recovery in AIS patients**.

Based on ROC curve analysis, Lp-PLA_2_ combined with IMA performed better than Lp-PLA_2_ or IMA alone in predicting the efficiency of rTMS plus GDLMI in promoting the cognitive and neurological function recovery in AIS patients (*p* < 0.05) (Table 6 and Fig. 3).

**Table 6.** **Predictive efficacy of Lp-PLA_2_ and IMA for efficiency of rTMS plus GDLMI in promoting the cognitive and neurological function recovery in AIS patients**.

	Predictive efficacy for cognitive function recovery					Predictive efficacy for neurological function recovery				
	Sensitivity (%)	Specificity (%)	Youden index	AUC	95% CI	Sensitivity (%)	Specificity (%)	Youden index	AUC	95% CI
Lp-PLA_2_	85.50	81.23	0.67	0.867	0.735–0.998	83.24	81.27	0.64	0.825	0.781–0.948
IMA	79.07	80.12	0.59	0.776	0.597–0.954	81.19	80.33	0.62	0.814	0.762–0.927
Combination	90.90	83.03	0.74	0.964	0.900–1.020	86.71	80.11	0.67	0.921	0.876–1.000

AUC, area under the curve.

## Discussion

Ginkgolides are the major component of GDLMI, which can effectively inhibit platelet activating factor-induced thrombosis and platelet aggregation, alleviate ischemic brain damage through the signal transducer and activator of transcription 3 pathway, and reduce the level of oxidative stress, thereby maintaining the cognitive and neurological function recovery in AIS patients [11]. rTMS is a non-invasive and effective therapy that induces electrical currents in cerebral tissue, promoting dopamine release and enhancing cortical excitability, thereby modulating cerebral blood flow and metabolism [12, 13]. In this study, the cognitive and neurological functions of AIS patients recovered to a certain extent after treatment using rTMS combined with GDLMI, but cognitive and neurological impairment was still present in some cases. Possibly, some related indices affected the efficiency of the recovery process.

Lp-PLA_2_, also known as platelet-activating factor acetylhydrolase, is secreted by macrophages, lymphocytes, mast cells, and platelets, which can bind various lipoproteins dominated by low-density lipoprotein through interacting with apolipoprotein B in the blood circulation [14, 15]. Lp-PLA_2_ can produce lysophosphatidylcholine *via* hydrolyzing oxidized phospholipids, produce oxidatively modified low-density lipoproteins *via* oxidizing non-esterified fatty acids, and release pro-inflammatory and pro-atherosclerotic metabolites to the circulation [16]. In the pathological state, Lp-PLA_2_ upregulates cytokine and adhesion factor levels, thereby disrupting vascular endothelial homeostasis [17]. As a sensitive index for assessing acute ischemia time, IMA has N-terminal binding sites altered under the action of acid and reactive oxygen species, which weakens its ability to bind metal ions [18, 19, 20]. When brain tissue is under hypoxic and ischemic states, excessive free radicals are generated, followed by a cascade reaction that extends over time [20, 21, 22]. Consequently, IMA is excessively generated during the progression of AIS.

In this study, the levels of Lp-PLA_2_ and IMA were significantly higher in patients with neurological impairment than those in patients with normal neurological function after rTMS combined with GDLMI, and Lp-PLA_2_ and IMA levels were linearly correlated with the efficiency of the combined therapy in promoting neurological function recovery. These findings suggest that Lp-PLA_2_ and IMA are associated with the process of neurological function recovery [23]. However, the correlation between IMA and cognitive impairment in AIS remains largely unexplored. In this investigation, the potential association between Lp-PLA_2_ and IMA levels and cognitive function recovery was further analyzed by grouping the patients based on the improvement degree in cognitive function. The results revealed that elevated levels of Lp-PLA_2_ and IMA were observed in patients exhibiting cognitive impairment. Consequently, these findings suggest that Lp-PLA_2_ and IMA levels are correlated with cognitive function recovery in patients with AIS [24].

Furthermore, our findings indicated that Lp-PLA_2_ and IMA were associated with treatment efficiency. The combination of rTMS and GDLMI demonstrated superior predictive efficacy compared to rTMS or GDLMI alone. Therefore, the efficiency of rTMS combined with GDLMI in promoting cognitive and neurological function recovery in AIS patients can be predicted based on the levels of Lp-PLA_2_ and IMA [7]. This prognostic information enables the early implementation of targeted interventions to optimize cognitive and neurological function recovery in these patients.

Nevertheless, this study has several limitations. First, the data were derived from a single medical center, potentially limiting the generalizability of the findings. Second, the sample size (n = 120) is relatively small. Additionally, our methodology was confined to comparing the scores of cognitive and neurological impairments before and after treatment. Without a negative control group for comparison, the efficacy assessment of rTMS plus GDLMI on AIS may be affected by the natural history of the disease itself, the placebo effect, and other treatments that may affect the development and prognosis of the disease. Consequently, the results may have bias. Further multicenter studies with larger sample sizes and a negative control group are needed to confirm our findings.

## Conclusions

In conclusion, rTMS combined with GDLMI can contribute to the cognitive and neurological function recovery in AIS patients. Serum levels of Lp-PLA_2_ and IMA may serve as independent predictive factors for the efficiency in promoting the cognitive and neurological function recovery. Therefore, the levels of Lp-PLA_2_ and IMA should be early monitored to predict the efficacy in cognitive and neurological function recovery and to improve the clinical efficacy.

## Availability of Data and Materials

The data and materials are available from the corresponding author upon reasonable request.
